# Impact of bariatric surgery on cerebral vascular reactivity and cognitive function: a non-randomized pilot study

**DOI:** 10.1186/s40814-020-00569-2

**Published:** 2020-02-13

**Authors:** Wesley J. Tucker, Binu P. Thomas, Nancy Puzziferri, T. Jake Samuel, Vlad G. Zaha, Ildiko Lingvay, Jaime Almandoz, Jing Wang, Edward A. Gonzales, R. Matthew Brothers, Michael D. Nelson

**Affiliations:** 1 Applied Physiology and Advanced Imaging Laboratory, Department of Kinesiology, University of Texas at Arlington, Science & Engineering Innovation & Research Building, 701 S. Nedderman Drive, Room 105, Arlington, TX 76019 USA; 2 Department of Nutrition & Food Sciences, Texas Woman’s University, Houston, TX USA; 3 Advanced Imaging Research Center, University of Texas Southwestern Medical Center, Dallas, TX USA; 4 Department of Bioengineering, University of Texas at Arlington, Arlington, TX USA; 5 Department of Surgery, Oregon Health & Science University, Portland, OR USA; 6 Division of Cardiology, University of Texas Southwestern Medical Center, Dallas, TX USA; 7 Division of Endocrinology, Diabetes, and Metabolism, University of Texas Southwestern Medical Center, Dallas, TX USA; 8 College of Nursing and Health Innovation, University of Texas at Arlington, Arlington, TX USA

**Keywords:** BOLD MRI, Hypercapnia, Middle cerebral artery dilation, Obesity, Sleeve gastrectomy

## Abstract

**Background:**

Bariatric surgery is an effective long-term weight loss strategy yielding improvements in neurocognitive function; however, the mechanism(s) responsible for these improvements remains unclear. Here, we assessed the feasibility of using magnetic resonance imaging (MRI) to evaluate whether cerebral vascular reactivity (CVR) is impaired in severely obese bariatric surgery candidates compared with normal weight healthy controls and whether CVR improves following bariatric surgery. We also investigated whether changes in CVR were associated with changes in cognitive function.

**Methods:**

Bariatric surgery candidates (*n* = 6) were compared with normal weight healthy controls of a similar age (*n* = 10) at baseline, and then reassessed 2 weeks and 14 weeks following sleeve gastrectomy bariatric surgery. Young reference controls (*n* = 7) were also studied at baseline to establish the range of normal for each outcome measure. Microvascular and macrovascular CVR to hypercapnia (5% CO_2_) were assessed using blood-oxygen-level-dependent (BOLD) MRI, and changes in the middle cerebral artery (MCA) cross-sectional area, respectively. Cognitive function was assessed using a validated neurocognitive software.

**Results:**

Compliance with the CVR protocol was high. Both macro- and micro-cerebrovascular function were highest in the young reference controls. Cognitive function was lower in obese bariatric surgery candidates compared with normal weight controls, and improved by 17% at 2 weeks and 21% by 14 weeks following bariatric surgery. To our surprise, whole-brain CVR BOLD did not differ between obese bariatric surgery candidates and normal weight controls of similar age (0.184 ± 0.101 vs. 0.192 ± 0.034 %BOLD/mmHgCO_2_), and did not change after bariatric surgery. In contrast, we observed vasoconstriction of the MCA during hypercapnia in 60% of the obese patients prior to surgery, which appeared to be abolished following bariatric surgery. Improvements in cognitive function were not associated with improvements in either CVR BOLD or MCA vasodilation after bariatric surgery.

**Conclusions:**

Assessing CVR responses to a hypercapnic challenge with MRI was feasible in severely obese bariatric patients. However, no changes in whole-brain BOLD CVR were observed following bariatric surgery despite improvements in cognitive function. We recommend that future large trials assess CVR responses to cognitive tasks (rather than hypercapnia) to better define the mechanisms responsible for cognitive function improvements following bariatric surgery.

## Background

Obesity has reached epidemic proportions worldwide and has led to a parallel rise in the prevalence of type 2 diabetes mellitus, together with an elevated risk of cardiovascular disease and death [1]. Importantly, obesity is associated with cognitive dysfunction and is a primary precursor for several neurocognitive and cerebrovascular diseases, including Alzheimer’s, dementia, and stroke [2–7]. While the exact mechanism responsible for the aforementioned conditions remains incompletely understood, cerebral vascular dysfunction is believed to be a major contributor [8–11]. Indeed, impaired cerebral vascular function has been linked with cognitive decline in healthy aging and is present in many disease states with known cognitive impairments including hypertension, heart failure, Alzheimer’s disease, and dementia [12–15]. Moreover, reduced cerebral vascular function both predicts, and contributes to, future stroke events [16–18].

The cerebral vasculature is tightly regulated by arterial carbon dioxide (CO_2_) tension such that under normal physiological conditions, hypercapnia increases cerebral blood flow (CBF) and hypocapnia reduces CBF [19, 20]. As such, indicators of the relative change in CBF, or cerebral blood velocity, per unit change in the partial pressure of CO_2_, are commonly used as an index of cerebral vascular reactivity (CVR) [21, 22]. A reduction in CVR to a vasodilatory stimulus like CO_2_ represents abnormal cerebral vascular health [21], with a diminished CVR response present in a number of clinical conditions including diabetes [23], hypertension [24], and carotid artery disease [22]. A blunted CVR response is also associated with an increased risk of stroke and mortality [18, 25].

CVR has been shown to be attenuated in obese individuals relative to lean, age-matched controls [10, 11]. However, these studies assessed CVR in response to hypercapnic challenges via changes in cerebral blood velocity by transcranial Doppler ultrasound of the middle cerebral artery (MCA). This method of assessing CVR relies on the assumption that the cross-sectional area (CSA) of the MCA does not change during hypercapnia [26], and therefore, changes in MCA velocity reflect changes in cerebral vascular perfusion. However, recent findings, using more sophisticated and sensitive imaging modalities, indicate that this assumption may not be true [21, 27–29]. Alternatively, CVR to hypercapnia can instead be assessed using blood-oxygen-level-dependent (BOLD) magnetic resonance imaging (MRI)—providing a whole-brain microvascular assessment of oxygenation changes due to increases in CBF in response to CO_2_—and MCA CSA changes by anatomical MRI—providing a measure of cerebral vascular health under the assumption that a vasodilative hypercapnic stimulus would increase MCA CSA. However, given the space limitations associated with BOLD and MCA CSA MRI assessment techniques, the feasibility and tolerability of using these assessment techniques in severely obese individuals is unclear.

Bariatric surgery is an effective long-term weight loss strategy, with dramatic improvements in hypertension and diabetes [30, 31]. These favorable improvements translate into meaningful clinical outcomes, including improvements in neurocognitive function [2, 32–35], Alzheimer’s disease risk [36, 37], and stroke events [37–40]. However, exactly how bariatric surgery improves neurocognitive outcomes remains to be elucidated. Emerging evidence suggests that bariatric surgery beneficially modulates a number of molecular culprits that lead to vascular dysfunction, including attenuation of oxidative stress and decreased levels of systemic inflammation [41, 42], and improves measures of peripheral vascular function (i.e. flow-mediated dilation) [42]. As such, improvements in neurocognitive outcomes following bariatric surgery may be mediated through changes in cerebral vascular function.

## Methods

### Study objectives

The primary objective of this pilot study was to determine the feasibility of our BOLD and MCA CSA MRI imaging techniques for assessing CVR in severely obese individuals. A secondary objective was to assess the impact of obesity on CVR, independent of age. A tertiary objective was to evaluate the short (within 2 weeks) and longer term (14 weeks) effect of bariatric surgery on CVR, and whether these effects were related to changes in cognition.

### Experimental design and protocols

This was a non-randomized observational pilot study. All experimental visits took place in the UT Southwestern Advanced Imaging Research Center (Dallas, TX). To accomplish the primary and secondary objectives, we compared CVR using BOLD and anatomical imaging of the MCA before and after CO_2_ inhalation across three groups: (1) obese pre-bariatric surgery candidates (1–10 days prior to surgery), (2) healthy normal weight (BMI < 25 kg/m^2^) controls of a similar age to the bariatric candidates (“age-matched healthy controls”), and (3) healthy young normal weight (BMI < 25 kg/m^2^) reference controls. To accomplish the tertiary objective, CVR was reassessed in the obese bariatric surgery candidates at 10–20 days post-surgery (defined as “2 weeks” here forward), and 10–16 weeks post-surgery (defined as “14 weeks” here forward). Young and older normal weight controls were only required to participate in 1 experimental visit. Prior to each experimental visit, all participants refrained from alcohol and caffeine for a minimum of 12 h. In addition, participants held medications and did not consume any food after waking up on the morning of each study visit.

### Ethical approval and research subjects

The study was approved by the Institutional Review Board for research involving human subjects at the University of Texas Southwestern Medical Center (STU 042016-080). All subjects gave informed written consent prior to participation in the study.

**Fig. 1:**
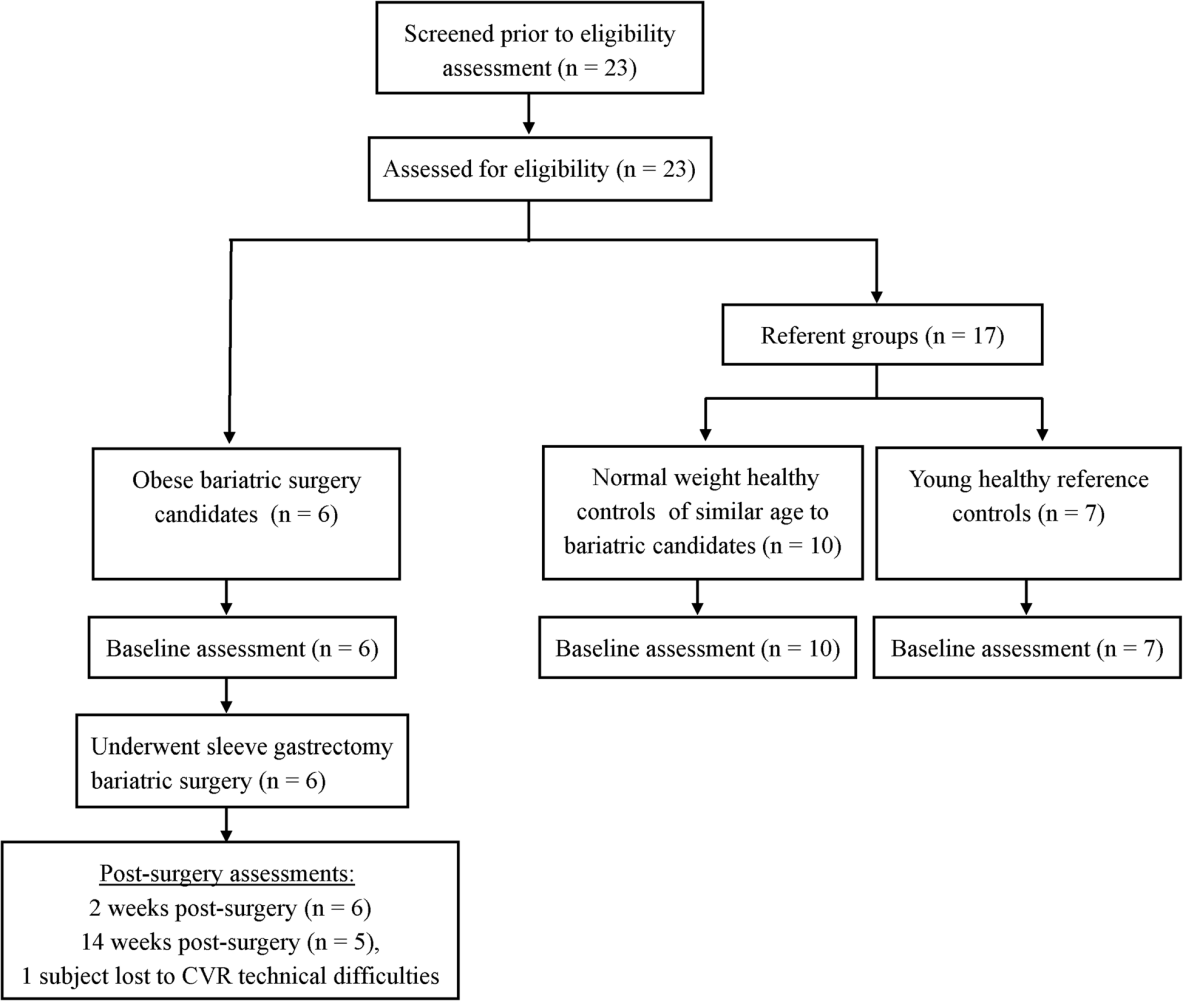
CONSORT flow diagram of study participants

Six obese bariatric surgery candidates (52 ± 10 years, 5 females, 41.9 ± 3.9 kg/m^2^), 10 normal weight controls of similar age (48 ± 6 years, 8 females, 22.8 ± 1.9 kg/m^2^), and seven young reference controls (24 ± 5 years, 2 females, 23.1 ± 1.9 kg/m^2^) participated in this study (Fig. 1). All subjects had at minimum a high school education. Further subject demographic information including the presence of co-morbidities and medication use can be found in Table 1. All obese surgery patients included in this study were recruited from and successfully underwent sleeve gastrectomy surgery at the UT Southwestern Multidisciplinary Surgery Clinic (Dallas, TX) between October 2016 and August 2017. Obese pre-bariatric surgery patients were excluded for significant anemia (hemoglobin < 10 mg/dl), abnormal renal function (serum creatinine above normal limit for age and sex), chronic respiratory conditions (chronic obstructive pulmonary disease or asthma), pregnancy, waist circumference > 65 in., incretin mimetic or dipeptidyl peptidase IV inhibitor use during the last 3 months, or contraindications to MRI. To assess the effect of obesity on cerebrovascular health, healthy normal weight controls of a similar age and gender were recruited to compare against obese pre-bariatric surgery patients. In addition, young reference controls were studied to serve as a control group to verify the effect of age (independent of BMI) on cerebrovascular health. Control subjects (both young reference and those age-matched to bariatric patients) were recreationally active, non-smokers with no history of cardiovascular (e.g. hypertension, type 2 diabetes) or cerebrovascular diseases (e.g. history of stroke, transient ischemic attack), major psychiatric or neurological disorders, or contraindications to MRI.

**Table 1:** Subject characteristics

	Obese pre-bariatric surgery patients	Age-matched healthy controls	Young reference controls
*N*	6	10	7
Gender (M, F)	(1, 5)	(2, 8)	(5, 2)
Age (years)	52 ± 10	48 ± 6	24 ± 5
Weight (kg)	118.1 ± 18.0	66.3 ± 7.3	70.3 ± 8.6
Body mass index (kg/m^2^)	41.9 ± 3.9	22.8 ± 1.9	23.1 ± 1.9
Systolic BP (mmHg)	121 ± 7	116 ± 10	115 ± 11
Diastolic BP (mmHg)	77 ± 8	75 ± 6	71 ± 8
Comorbidities			
Hypertension	5 (83%)	−	–
Hypercholesterolemia	4 (67%)	−	−
Diabetes	2 (33%)	−	−
Hypothyroidism	3 (50%)	2 (20%)	−
Anxiety/depression	4 (67%)	2 (20%)	−
Medications			
ACE inhibitor	4 (67%)	−	−
Beta-blocker	2 (33%)	−	−
Statin	1 (17%)	−	−
Biguanide	2 (33%)	−	−
Levothyroxine	3 (50%)	2 (20%)	−
SSRIs	3 (50%)	2 (20%)	−

Prevalence of comorbidity and medication use displayed as n (% of group). All other data displayed as Mean ± SD. *ACE*: angiotensin-converting enzyme, *BP*: blood pressure, *SSRIs*: selective serotonin reuptake inhibitors

### Anthropometrics and blood pressure

At each visit, height and weight were measured with a standard stadiometer and scale (Health-O-Meter, Sun Beam Inc., Boca Raton, FL, USA) for calculation of body mass index (BMI) in kg/m^2^. Waist circumference measurements were taken at the level of the umbilicus using a standard Gullick tape measure. Three seated blood pressure measurements were averaged to obtain resting systolic and diastolic blood pressures, with a 2-min break in between each measurement (Welch Allyn, Skaneateles, NY, USA).

### MRI experiments

All MRI scans were performed on a 3-T Philips Achieva system (Philips Medical Systems, Best, The Netherlands), using an 8-channel receive-only head coil. A body coil was used for radiofrequency transmission. Foam padding was placed around the head to minimize motion during MRI scan acquisition. CVR response to CO_2_ was assessed using a hypercapnic challenge (inhalation of 5% CO_2_ mixed with 21% O_2_ and 74% N_2_) as described in detail previously [43, 44]. In brief, alternating blocks of room air (1 min) and hypercapnia (1 min) were inhaled by the subject while BOLD images were continuously acquired for 7 min. Compared with a long-duration hypercapnia paradigm, this alternating 1-min off, 1-min on CO_2_ protocol has been shown to improve subject comfort while maintaining high data quality [44]. The imaging parameters were TR/TE/FA = 1500 ms/30 ms/60°, voxel size = 3.0 × 3.0 × 5.0 mm^3^, FOV = 240 × 240 mm^2^, 29 slices, and thickness = 5 mm. The air/gas mixture was delivered to subjects using a Douglas bag and switching between room air and CO_2_ was achieved via a valve connected to the bag. The air/gas mixture was delivered to the subject via a mouthpiece with a nose clip placed to prevent nasal breathing during the protocol. Partial pressure of exhaled CO_2_ (P_ET_CO_2_) was measured using a calibrated capnographic monitor (Capnograd, Model 1265, Novametrix Medical Systems, Wallingford, CT, USA). P_ET_CO_2_ is a strong stimulus for vasodilatory input to the brain. Other physiological parameters including breath rate, heart rate and arterial oxygenation were also continuously measured during each MRI scan using a physiology monitor (MEDRAD Inc., Pittsburgh, PA).

MCA vasodilatory response to hypercapnia was assessed by measuring MCA cross-sectional area (CSA) during a 3-min baseline (room air) and then again following 3 min of continuously breathing the hypercapnic air/gas mixture (5% CO_2_ mixed with 21% O_2_ and 74% N_2_). To locate and quantify the MCA CSA, a 3D time-of-flight sequence was used to select the location on the M1 segment of the right or left MCA, followed by a T2-weighted fast spin echo sequence (8 slices, TR/TE/FA = 3000/100/120°, voxel dimensions = 0.4 × 0.4 × 2.0 mm^3^) gated to mid-diastole [21]. Selection of either the right or left MCA was dictated by individual anatomy and image quality. In bariatric patients only, the side of the MCA (left or right) used during pre-bariatric surgery visits was also used at follow-up visits (2 weeks and 14 weeks post-surgery) to ensure consistency within subject. P_ET_CO_2_, breath rate, heart rate, and arterial saturation were continuously measured during this scan using the same physiology monitor described above.

In addition, a T1-weighted high-resolution image was acquired using the Magnetization-Prepared-Rapid-Acquisition-of-Gradient-Echo (MPRAGE) sequence (voxel size = 1 × 1 × 1 mm^3^, scan duration = 4 min). This image was used to normalize the BOLD images from subject space to Montreal Neurological Institute template space.

### MRI data processing

CVR data were processed using procedures previously described [43–46]. First, the time lag between P_ET_CO_2_ time-course and global BOLD response was calculated by shifting the P_ET_CO_2_ time-course one second at a time until maximum cross correlation was obtained. An important note, the BOLD time-course always lags behind the P_ET_CO_2_ time-course because of the transit time required for blood in the lungs (where P_ET_CO_2_ is measured) to travel to the heart and then be pumped to the brain for brain vessels to react to the higher CO_2_ level (when the BOLD change occurs). Thereafter, the shifted P_ET_CO_2_ was used as a regressor in the Statistical Parametric Mapping’s General Linear Model to estimate the CVR on a voxel-by-voxel basis. The CVR has units of % BOLD signal change per mmHg of P_ET_CO_2_ change (% BOLD/mmHg CO_2_). Mean CVR for the whole brain was also measured for each subject using a mask in Montreal Neurological Institute template space (obtained from the WFU_pickatlas software) [47].

The MCA CSA was measured manually in duplicate by a blinded observer using commercially available imaging software (CVI^42^, Circle Cardiovascular Imaging Inc., Canada). The data from duplicate measurements were averaged and reported. The coefficient of variation (% CV) between these duplicate measurements of MCA CSA was 2.8 ± 2.2% during baseline and 3.7 ± 2.4% during hypercapnia. The reported CSA during hypercapnia is the CSA measured after 3 min of continuous inhalation of 5% CO_2_ and this is reported along with absolute (mm^2^) and percent (%) change from baseline (room air). All other physiological variables (P_ET_CO_2_, breath rate, heart rate) reported during hypercapnia correspond to these time points.

### Cognitive function assessment

Cognitive function was assessed in bariatric patients and normal weight controls of a similar age at each experimental visit using the validated IntegNeuro™ computerized neurocognitive test battery system (Brain Resource Company Ltd, Sydney, Australia). This neurocognitive test assesses multiple domains of cognition with strong validity against traditional paper and pencil neuropsychological tests examining the same cognitive constructs [48]. Domains assessed by the test include response speed, impulsivity, attention, information processing, memory, executive function, and emotion identification. IntegNeuro™ uses different task versions for repeated testing sessions to minimize the effect of familiarization and practice effects, with test-retest reliability measures showing high levels of consistency [49, 50]. This test battery system is also sensitive to cognitive impairments in individuals with elevated BMI and bariatric surgery candidates [32, 33, 35, 51]. Cognitive function scores range from 1 to 10 with higher scores indicating better cognitive function. Cognitive function was only assessed in 5 bariatric patients as the neurocognitive software only became available for use after completion of our first bariatric patient.

### Evaluation of feasibility outcomes

To evaluate the feasibility of our BOLD and MCA CSA MRI imaging techniques to assess CVR responses to hypercapnia in severely obese individuals, we recorded subject compliance with these imaging protocols prior to and following bariatric surgery. Completion of both BOLD and MCA CSA MRI imaging protocols while undergoing the hypercapnic challenge was considered 100% compliance.

### Sample size

The primary endpoint of this study was to assess the feasibility of our BOLD and MCA CSA MRI imaging techniques to assess CVR responses to hypercapnia in severely obese individuals; as such, a formal sample size calculation was not performed. To evaluate the feasibility of the present study, we considered it necessary to test our BOLD and MCA CSA MRI imaging techniques repeatedly on a minimum of five severely obese bariatric surgery patients.

### Statistical analysis

All statistical analyses were performed using Statistical Package for Social Sciences (version 22, IBM SPSS, Armonk, NY). Descriptive data are presented as mean ± SD. Comparative data are presented as mean differences and 95% confidence intervals. All data were evaluated for normality with Shapiro-Wilk tests and Q-Q plots. Unpaired *t* tests were conducted to assess mean differences in whole-brain CVR and MCA vasodilation by age (young reference controls versus age-matched healthy controls). Thereafter, unpaired *t* tests were used to assess mean differences in whole-brain CVR, MCA vasodilation and cognitive function by BMI (age-matched healthy controls versus obese pre-bariatric surgery patients). Paired *t* tests were used to assess changes in MCA CSA and other physiological variables in response to hypercapnia within condition (baseline versus hypercapnia). A one-way repeated measures ANOVA assessed changes in whole-brain CVR, MCA vasodilation and cognitive function after bariatric surgery (2 weeks and 14 weeks post-surgery). Holm-Sidak tests were used when appropriate. Pearson correlations were used to assess associations between ∆ whole-brain CVR, ∆ MCA vasodilation, and ∆ cognitive function scores following bariatric surgery.

## Results

### Subject demographics

Subject characteristics are presented in Table 1. By design, the obese bariatric surgery patients had higher body weight and BMI. This group of subjects also reported higher rates of hypertension and hypercholesterolemia than normal weight controls of similar age (“age-matched healthy controls”) and young reference controls. Obese pre-bariatric surgery patients and age-matched healthy controls had more cases of hypothyroidism and anxiety/depression compared with young reference controls. As a result, the use of levothyroxine and selective serotonin reuptake inhibitors were more prevalent in obese pre-bariatric surgery patients and age-matched healthy controls.

### Subject compliance

CVR imaging techniques were well tolerated by severely obese individuals before and after bariatric surgery with a 92% overall assessment completion rate (33 of 36 imaging visits fully completed). All six bariatric surgery subjects completed the CVR BOLD imaging protocol for all visits (100% completion rate). CVR data from one bariatric patient collected during the 14-week post-surgery visit was not usable due to experimental error/equipment malfunction. However, this was due to investigator error not due to subject compliance. MCA CSA was only collected in 5 bariatric patients at all visits (83% completion rate) as one bariatric patient requested a shortened MRI protocol (assessment of whole-brain BOLD CVR but not MCA CSA) due to mild lower back discomfort associated with lying in the MRI.

## Effect of obesity on cerebral vascular reactivity

### Blood-oxygen-level-dependent MRI

**Fig. 2:**
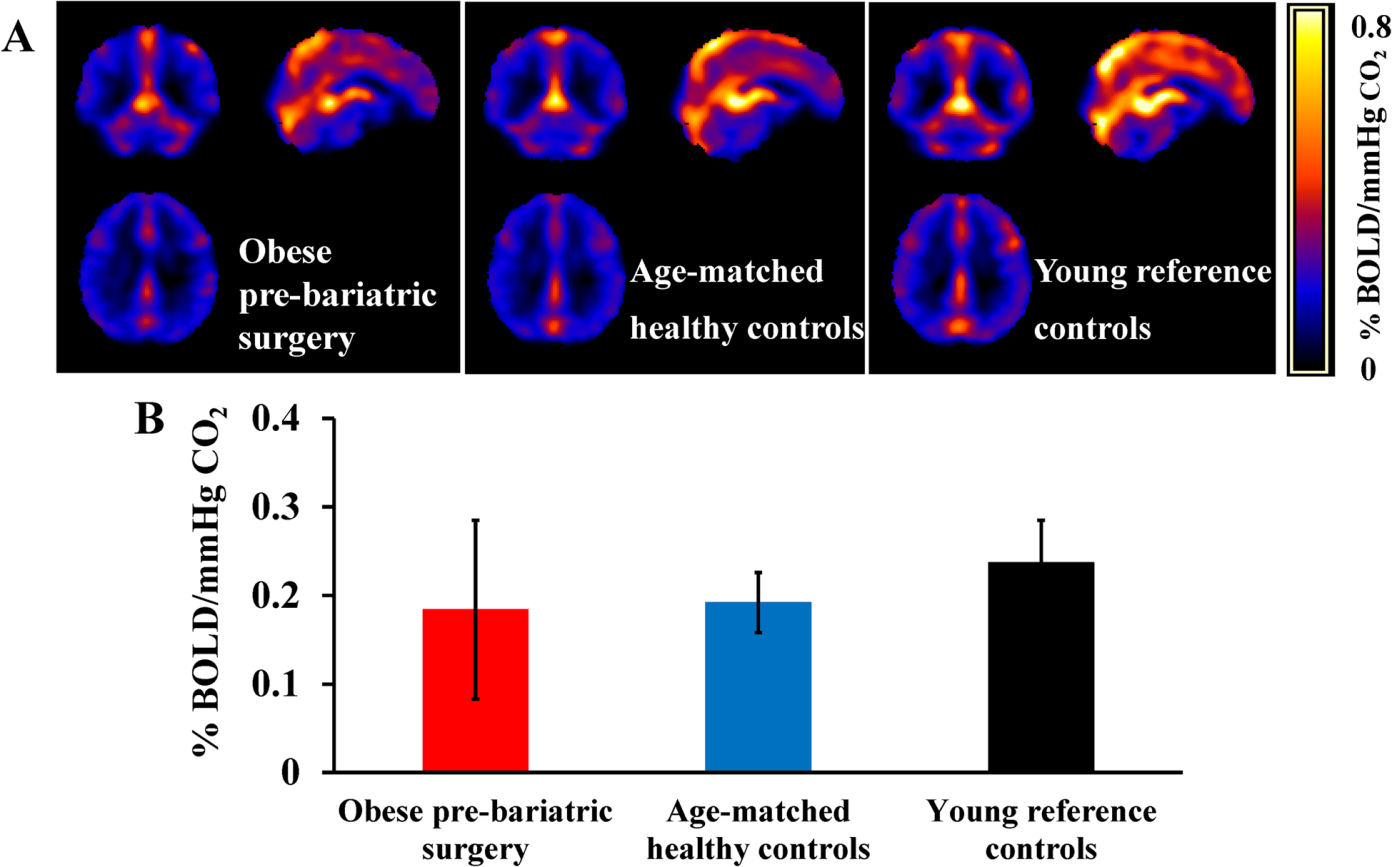
Cerebral vascular reactivity (CVR) measured with whole brain blood-oxygen-level dependent (BOLD) MRI responses to hypercapnic stimulus (5% CO_2_) in obese pre-bariatric surgery patients prior to surgery, age-matched healthy controls, and young reference controls. **a** BOLD maps, showing regional changes in blood oxygenation in response to hypercapnia. **b** Whole-brain BOLD CVR responses by group. Data are mean ± SD

As expected, CO_2_-inhalation increased P_ET_CO_2_, breath rate and heart rate during hypercapnia (Supplemental Table 1). As illustrated in Fig. 2, whole-brain CVR was higher in young reference controls (0.237 ± 0.048 %BOLD/mmHg CO_2_) compared with the age-matched healthy controls (0.192 ± 0.034 %BOLD/mmHg CO_2_, mean difference 0.045 %BOLD/mmHg CO_2_; 95% CI 0.003 to 0.087). However, we did not observe an obesity-independent effect on whole-brain CVR (i.e. obese patients did not differ from normal weight control of similar age) (Fig. 2b).

### Middle cerebral artery (MCA) vasodilation

**Fig. 3:**
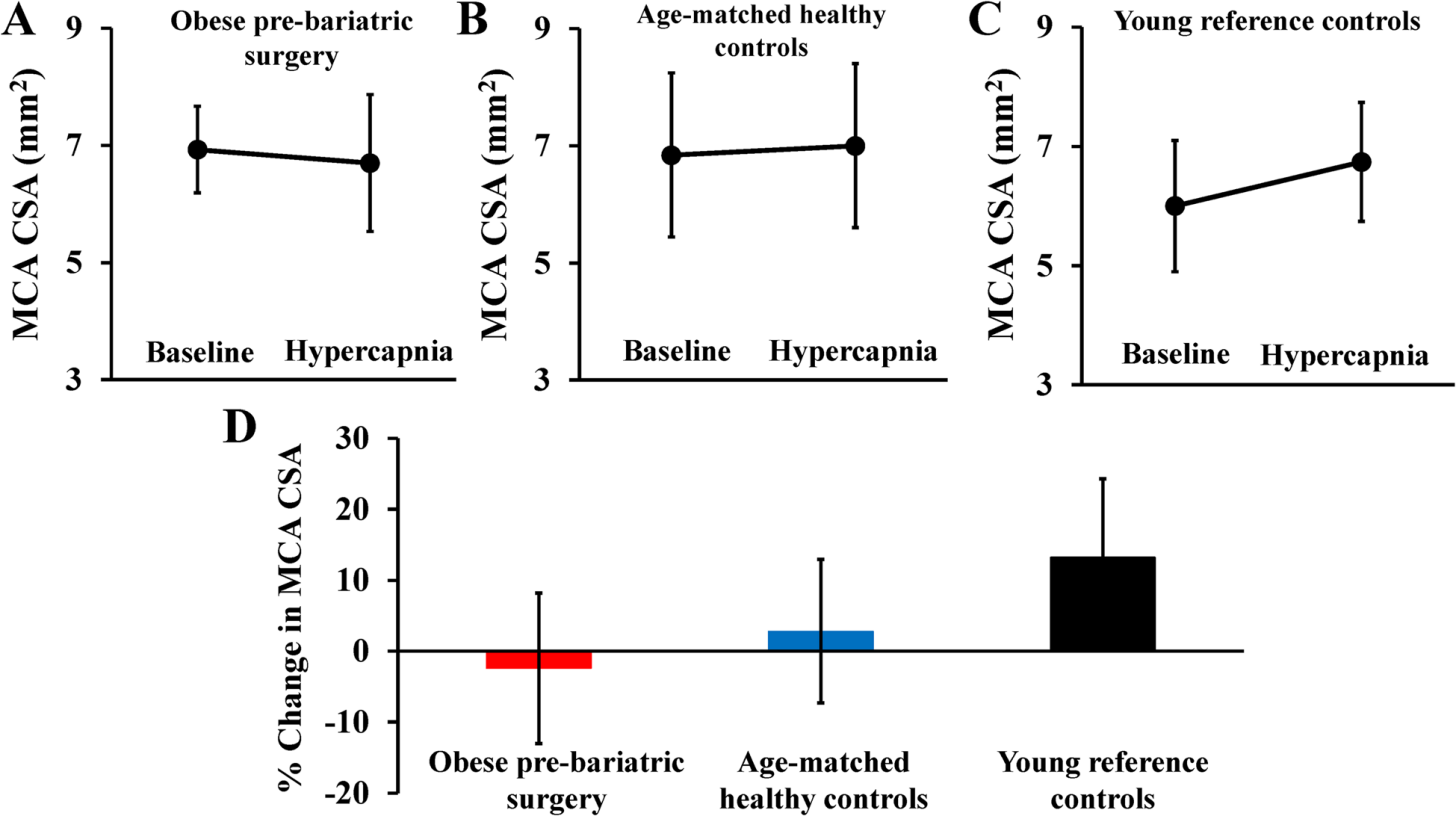
Middle cerebral artery (MCA) cross-sectional area (CSA) responses to 3 min of hypercapnic stimulus in **a** obese pre-bariatric surgery patients, **b** age-matched healthy controls, **c** young reference controls, and **d** mean percent changes in CSA by group. Data are mean ± SD

Consistent with an emerging body of evidence, MCA CSA increased in response to hypercapnia in young reference controls (6.0 ± 1.1 to 6.7 ± 1.0 mm^2^, mean change + 0.7 mm^2^; 95% CI 0.2 to 1.3 mm^2^, Fig. 3c). In contrast, MCA CSA did not increase in response to hypercapnia in the obese bariatric surgery patients prior to surgery (6.9 ± 0.7 to 6.7 ± 1.2 mm^2^, mean change − 0.2 mm^2^; 95% CI − 0.7 to 0.1 mm^2^, Fig. 3a), or in the age-matched healthy controls (6.8 ± 1.4 to 7.0 ± 1.4 mm^2^, mean change +0.2 mm^2^; 95% CI − 0.3 to 0.7 mm^2^, Fig. 3b). In fact, we observed vasoconstriction of the MCA in response to hypercapnia in 60% of the obese pre-bariatric surgery patients, indicating marked macrovascular dysfunction (Fig. 3d). Hemodynamic and end-tidal responses to the hypercapnia are reported in Supplemental Table 2, with no major differences observed between groups.

## Effect of bariatric surgery

The median duration post-surgery for follow-up experimental visits in bariatric patients was 16 days (range 12 to 20 days) post-surgery (2 weeks post-surgery visit) and 98 days (range 72 to 110 days) post-surgery (14 weeks post-surgery). One patient reduced their dosage of metformin from 1000 mg twice/day at baseline and 2 weeks post-surgery to 500 mg twice/day by 14 weeks. No other changes in medications were reported by the bariatric patients.

As outlined in Table 2, bariatric surgery resulted in expected reductions in body weight, BMI, and waist circumference. We also observed reductions in systolic blood pressure (SBP) and diastolic blood pressure (DBP) following surgery.

**Table 2:** Anthropometrics and blood pressure taken prior to, at 2 weeks, and at 14 weeks after bariatric surgery

	Pre-surgery	2 weeks post-surgery	Mean change 2 weeks (95% CIs)	14 weeks post-surgery	Mean change 14 weeks (95% CIs)
Body weight (kg)	118.1 ± 18.0	111.7 ± 16.0	−6.4 (−10.3, −2.5)	102.7 ± 18.5	−15.4 (−21.1, −9.7)
Body mass index (kg/m^2^)	41.9 ± 3.9	39.7 ± 3.8	−2.2 (−3.3, −1.0)	36.4 ± 4.7	−5.5 (−7.4, −3.7)
Waist circumference (cm)	125.3 ± 8.3	121.1 ± 8.0	−4.1 (−11.1, 2.8)	112.6 ± 9.2	−12.7 (−21.3, −4.0)
Systolic BP (mmHg)	121 ± 7	113 ± 5	−8 (−18, 3)	106 ± 10	−15 (−27, −3)
Diastolic BP (mmHg)	77 ± 8	72 ± 8	−5 (−12, 3)	70 ± 8	−7 (−13, −1)

*BP* blood pressure. *n* = 6. Data are mean ± SD. Differences from pre-surgery are presented as mean change with 95% confidence intervals (CIs)

### Blood-oxygen-level-dependent MRI in response to bariatric surgery

**Fig. 4:**
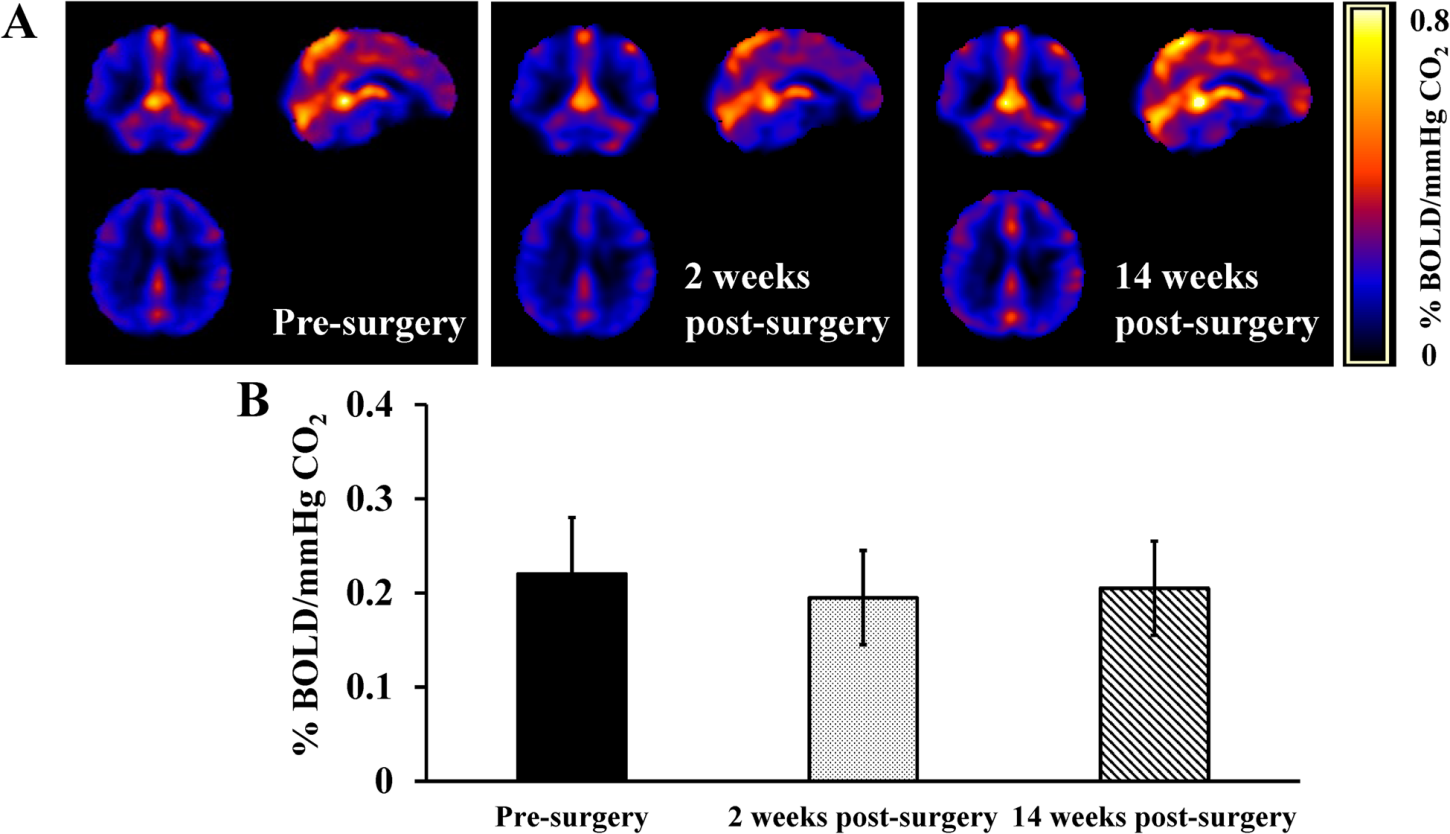
Cerebral vascular reactivity (CVR) measured with whole brain blood-oxygen-level dependent (BOLD) MRI responses to hypercapnic stimulus in obese bariatric surgery candidates before, and 2 weeks and 14 weeks after bariatric surgery. **a** BOLD maps and **b** mean whole-brain BOLD CVR responses to hypercapnic stimulus. Data are mean ± SD

Similar to the baseline measurements, the CO_2_-inhalation protocol increased P_ET_CO_2_, breath rate, and heart rate at each time point (Supplemental Table 3). However, we observed no improvement in whole-brain CVR after bariatric surgery (Main effect of time (F(2, 8) = 1.194, *p* = 0.35) (Fig. 4).

### Middle cerebral artery (MCA) vasodilation following bariatric surgery

**Fig. 5:**
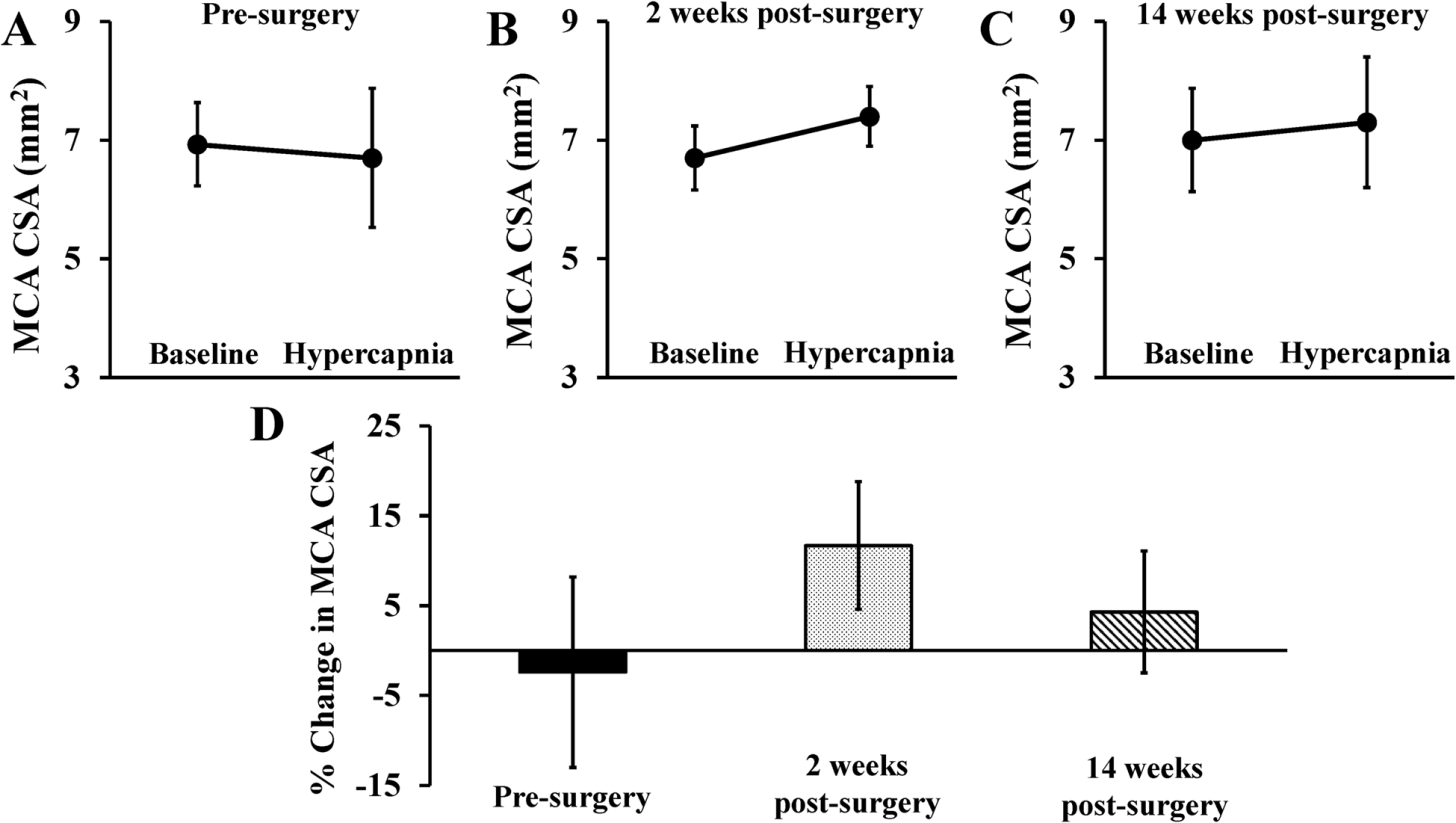
Middle cerebral artery (MCA) cross-sectional area (CSA) responses to 3 min of hypercapnic stimulus in obese bariatric surgery candidates before, and 2 weeks and 14 weeks after bariatric surgery. Mean absolute changes in CSA to hypercapnia at **a** pre-surgery, **b** 2 weeks post-surgery, and **c** 14 weeks post-surgery. **d** Mean percent changes in CSA by time point. Data are mean ± SD

Interestingly, MCA CSA increased in response to hypercapnia 2 weeks after surgery (6.7 ± 0.5 to 7.4 ± 0.5 mm^2^, mean change + 0.7 mm^2^; 95% CI 0.2 to 1.3 mm^2^), which could be indicative of acute improvements in MCA vasodilatory capacity (Fig. 5b). While the change in MCA vasodilation did not remain significant 14 weeks after surgery (7.0 ± 0.9 to 7.3 ± 1.1 mm^2^, mean change + 0.3 mm^2^; 95% CI − 0.3 to 1.0 mm^2^, Fig. 5c), it is important to note that none of the patients vasoconstricted with hypercapnia (an improvement from baseline). Hemodynamic and end-tidal responses to the hypercapnia are reported in Supplemental Table 4, with no major differences observed between time points (pre- and post-surgery).

### Cognitive function

**Fig. 6:**
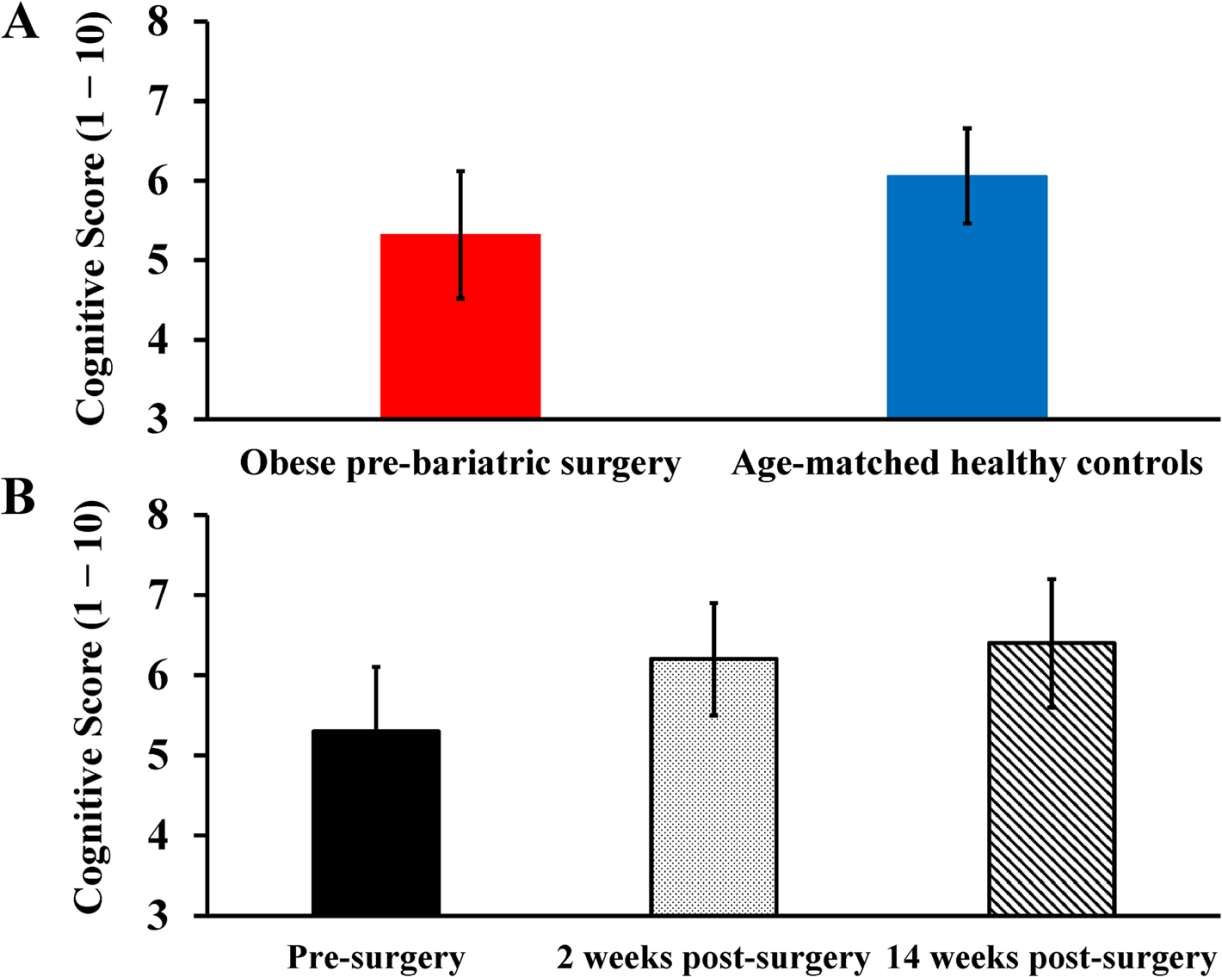
Cognitive function scores in obese pre-bariatric surgery patients compared with age-matched healthy controls (panel **a**). Change in mean cognitive function scores following bariatric surgery (panel **b**). Data are mean ± SD

Prior to surgery, cognitive function was lower in obese pre-bariatric surgery patients (5.3 ± 0.8) compared with age-matched healthy controls (6.0 ± 0.6, mean difference 0.7; 95% CI − 0.1 to 1.5), Fig. 6a). After bariatric surgery, we observed a significant main effect for time (F(2, 8) = 14.35, *p* = 0.002), with a 17% improvement in cognition at 2 weeks post-surgery (6.2 ± 0.7, mean change from pre-surgery +0.9; 95% CI 0.2 to 1.5) and a 21% improvement at 14 weeks post-surgery (6.4 ± 0.8, mean change from pre-surgery +1.1; 95% CI 0.6 to 1.6, Fig. 6b). However, these improvements in cognitive function were not associated with improvements in CVR BOLD (2 weeks: *r* = 0.15, *p* = 0.81; 14 weeks: *r* = 0.49, *p* = 0.39) or MCA vasodilation (2 weeks: *r* = 0.02, *p* = 0.98, 14 weeks: *r* = −0.06, *p* = 0.94) after bariatric surgery.

## Discussion

The main findings of this exploratory pilot study are as follows: First, the use of BOLD and MCA CSA MRI imaging techniques to assess CVR responses to hypercapnia was feasible and well tolerated in severely obese individuals. However, analysis of this data revealed that obesity was not associated with reduced whole-brain CVR or MCA vasodilatory responsiveness to hypercapnia when compared with normal weight controls of a similar age. In contrast, cerebral vascular vasodilator responsiveness appears to be largely dependent on age, with young reference controls demonstrating superior whole-brain CVR and MCA vasodilation. Interestingly, while microvascular whole-brain CVR to hypercapnia was not improved in the short (2 weeks) or longer term (14 weeks) following bariatric surgery, macrovascular MCA vasodilatory responsiveness to hypercapnia was improved. Lastly, while cognitive function tended to be reduced in obese bariatric surgery patients prior to surgery, and was improved following bariatric surgery, these improvements appear to be independent of changes in micro- or macrovascular function (assessed by cerebral vascular reactivity to a hypercapnic challenge).

### Effect of obesity on cerebral vascular reactivity

Contrary to previously published work [9–11], we did not observe impaired whole-brain CVR response to hypercapnia in obese individuals relative to normal weight individuals of a similar age. One potential explanation for this disparity could be attributed to differences in disease severity between studies. For example, Frosch et al. (2017) recently evaluated whether CVR to mild hypercapnia was different in obese/overweight individuals with and without insulin resistance compared with lean controls. Using high spatial resolution arterial spin labeling and a mild hypercapnic protocol (CO_2_ rebreathing to induce 5–7 mmHg increase in P_ET_CO_2_) the authors found that overweight/obese individuals had a lower CVR response to hypercapnia. However, in the overweight/obese individuals with insulin resistance, decreased insulin sensitivity was strongly associated with reduced CVR even after adjusting for BMI, suggesting that the metabolic consequences, and not obesity itself, may be driving the adverse effects on CVR. While we did not specifically evaluate insulin sensitivity in the present study, it is interesting to note that our most “at-risk” bariatric surgery candidate (according to medication use, BMI, and cardiovascular disease risk factors) had the lowest CVR by almost 2 standard deviations. In contrast, despite having an elevated BMI, the majority of patients we studied had normal blood pressures at baseline and were not being treated for metabolic or cardiovascular disorders. Future studies evaluating cerebral vascular reactivity changes with bariatric surgery may therefore benefit from including patients who are at higher risk.

This study also evaluated the influence of obesity on the vasodilatory capacity of the MCA during hypercapnia. Indeed, obese individuals have reduced endothelium-dependent vasodilation in peripheral large conduit arteries (brachial and femoral) in response to acetylcholine or shear stress suggesting a decrease in NO production or bioavailability in this population [52, 53]. Furthermore, patient populations with known impairments in peripheral large conduit artery endothelial function also have impaired CVR responses to hypercapnia [22–24], and these impairments are abolished after administration of an exogenous NO donor sodium nitroprusside [24]. Although the percent change in MCA CSA to hypercapnia was not statistically different between obese individuals and age-matched healthy controls, it is important to note that 60% of the obese bariatric surgery patients vasoconstricted during the CO_2_ challenge at baseline. This finding may reflect decreased NO bioavailability and cerebral vascular dysfunction. That these differences were observed in the MCA but not with whole-brain BOLD imaging may reflect important regional differences in cerebral vascular health that requires closer examination in future large-scale trials.

That age was related to whole-brain CVR and MCA vasodilatory responsiveness to hypercapnia, both confirm and extend prior investigations [21, 45, 54–56]. Indeed, while age-related reductions in CVR have been extensively documented [45, 54–57], only one study has investigated the effect of age on MCA vasodilatory responsiveness to hypercapnia [21]. Similar to our findings, Coverdale et al. (2017) showed that MCA CSA (as measured by 3 T MRI) increased consistently in young but not older adults in response to 6% CO_2_. Interestingly, when indomethacin is administered to block the prostaglandin mediated vasodilatory pathway, the differences in CVR between young and older adults are completely abolished, suggesting that the prostaglandin-mediated response to hypercapnia is impaired in older adults [58]. Future studies are needed to explore similar mechanistic pathways in older obese individuals.

### Effect of bariatric surgery on cerebral vascular reactivity and cognition

Our finding that bariatric surgery did not improve the whole-brain CVR response to hypercapnia is not surprising in the context that our obese volunteers had similar baseline whole-brain CVR responses compared with age-matched healthy controls. However, we observed a large and consistent improvement in MCA vasodilatory responsiveness to hypercapnia in all of the bariatric patients studied 2 weeks following bariatric surgery. Indeed, bariatric surgery elicits rapid improvements in peripheral large conduit artery endothelial function [59–61]. This improvement may be mediated in part by caloric restriction associated with a mandatory low-calorie, liquid diet. Short-term caloric restriction improves endothelial-dependent vasodilation through an increased release of nitric oxide and rapid improvement in insulin sensitivity and glycemic control [62, 63]. This interpretation would also be consistent with the vasoconstriction we observed in the majority of bariatric patients prior to surgery, when NO bioavailability was presumably lowest.

Bariatric surgery is known to improve cognitive function at 12 weeks and up to 3 years post-surgery [32–35, 64]. While the exact mechanism for cognitive improvement following bariatric surgery remains unknown, improvements in glycemic control and changes in satiety hormones have been implicated [64, 65]. To our knowledge, the current study is first to demonstrate cognitive function improvements in as little as 2 weeks following bariatric surgery, with this improvement being maintained at 14 weeks post-surgery. However, the improvement in cognitive function at 2 weeks and 14 weeks following bariatric surgery was not associated with improvements in cerebral vascular function, as measured by CVR and MCA vasodilation in response to CO_2_. That CVR to hypercapnia does not relate to changes in cognitive function may be an important distinction, however, and suggest that future studies may need to assess the BOLD response to cognitive tasks.

### Limitations and future studies

The current exploratory pilot study has several limitations. Our sample size was admittedly small, particularly in the obese bariatric surgery group. This was however a hypothesis-generating pilot study designed to assess the impact of bariatric surgery in a small number of subjects. A relatively small size allowed us to intensively recruit subjects given the resources and time frame. Our findings should therefore be viewed in this context. This study is also mechanistically limited. Based on the present results, future studies would benefit from incorporation of an NO donor and prostaglandin blockade to partition the independent contribution of these pathways on cerebral vascular responsiveness. Moreover, assessment of the BOLD response to a cognitive task, instead of hypercapnia, may help define the mechanism for cognitive function improvements.

## Conclusions

Assessing CVR responses to a hypercapnic challenge with MRI was feasible in severely obese bariatric patients. However, no changes in CVR responses to hypercapnia were observed following bariatric surgery despite improvements in cognitive function. We recommend that future large trials assess CVR responses to cognitive tasks (rather than hypercapnia) to better define the mechanisms responsible for cognitive function improvements following bariatric surgery.

## Supplementary information

**Additional file 1: Supplemental Table 1.** Physiological characteristics at baseline and during hypercapnia (HC) during acquisition of blood-oxygen-level-dependent (BOLD) images with MRI in obese pre-bariatric surgery patients, age-matched healthy controls, and young reference controls.**Supplemental Table 2.** Physiological characteristics at baseline and after 3 minutes of continuous hypercapnia (HC) during acquisition of middle cerebral artery (MCA) images with MRI in obese pre-bariatric surgery patients, age-matched healthy controls, and young reference controls. **Supplemental Table 3.** Physiological characteristics at baseline and after hypercapnia (HC) during acquisition of blood-oxygen-level-dependent (BOLD) images with MRI before bariatric surgery, and 2 weeks, and 14 weeks post-surgery. **Supplemental Table 3.** Physiological characteristics at baseline and after hypercapnia (HC) during acquisition of blood-oxygen-level-dependent (BOLD) images with MRI before bariatric surgery, and 2 weeks, and 14 weeks post-surgery. **Supplemental Figure 1.** Sagittal T2 baseline image (room air) of the left middle cerebral artery (MCA) of a representative subject. To assess MCA vasodilation capacity, the MCA cross-sectional area (CSA) was measured at baseline while subjects breathed room air and again after 3 min of hypercapnia (5% CO_2_, 21% O_2_, N_2_ balance) to calculate absolute and percent change in CSA.

## Data Availability

The datasets used and/or analyzed during the current study are available from the corresponding author on reasonable request.

## Abbreviations

BOLD: Blood-oxygen-level-dependent BMI Body mass index CBF Cerebral blood flow CO_2_ Carbon dioxide CSA Cross-sectional area CVR Cerebral vascular reactivity MCA Middle cerebral artery MRI Magnetic resonance imaging P_ET_CO_2_ Partial pressure of end-tidal carbon dioxide
