# Supplementary material for: Impact of bariatric surgery on cerebral vascular reactivity and cognitive function: a non-randomized pilot study
Source: Pilot Feasibility Stud. 2020 Feb 13;6:21. doi: 10.1186/s40814-020-00569-2 (PMC7017515; doi:10.1186/s40814-020-00569-2)
Supplement: Supplementary file 1 — Additional file 1: Supplemental Table 1. Physiological characteristics at baseline and during hypercapnia (HC) during acquisition of blood-oxygen-level-dependent (BOLD) images with MRI in obese pre-bariatric surgery patients, age-matched healthy controls, and young reference controls.Supplemental Table 2. Physiological characteristics at baseline and after 3 minutes of continuous hypercapnia (HC) during acquisition of middle cerebral artery (MCA) images with MRI in obese pre-bariatric surgery patients, age-matched healthy controls, and young reference controls. Supplemental Table 3. Physiological characteristics at baseline and after hypercapnia (HC) during acquisition of blood-oxygen-level-dependent (BOLD) images with MRI before bariatric surgery, and 2 weeks, and 14 weeks post-surgery. Supplemental Table 3. Physiological characteristics at baseline and after hypercapnia (HC) during acquisition of blood-oxygen-level-dependent (BOLD) images with MRI before bariatric surgery, and 2 weeks, and 14 weeks post-surgery. Supplemental Figure 1. Sagittal T2 baseline image (room air) of the left middle cerebral artery (MCA) of a representative subject. To assess MCA vasodilation capacity, the MCA cross-sectional area (CSA) was measured at baseline while subjects breathed room air and again after 3 min of hypercapnia (5% CO2, 21% O2, N2 balance) to calculate absolute and percent change in CSA. [file 40814_2020_569_MOESM1_ESM.docx]

**Supplemental Tables**

**Supplemental Table 1.** Physiological characteristics at baseline and during hypercapnia (HC) during acquisition of blood-oxygen-level-dependent (BOLD) images with MRI in obese pre-bariatric surgery patients, age-matched healthy controls, and young reference controls.

|  | **Obese pre-bariatric surgery** | | | **Age-matched healthy controls** | | | **Young reference controls** | | |
| --- | --- | --- | --- | --- | --- | --- | --- | --- | --- |
|  | **Baseline** | **HC** | **Δ (95% CI)** | **Baseline** | **HC** | **Δ (95% CI)** | **Baseline** | **HC** | **Δ (95% CI)** |
| Heart Rate (bpm) | 63 ± 15 | 69 ± 14 | 6 (4, 9) | 64 ± 15 | 70 ± 16 | 6 (3, 8) | 57 ± 3 | 63 ± 9 | 6 (2, 9) |
| Breath rate (b/min) | 9 ± 4 | 13 ± 4 | 4 (1, 6) | 8 ± 4 | 10 ± 4 | 2 (1, 4) | 10 ± 1 | 13 ± 6 | 3 (1, 8) |
| P_ET_CO_2_ (mmHg) | 45 ± 12 | 54 ± 10 | 9 (6, 12) | 41 ± 6 | 50 ± 4 | 9 (7, 11) | 42 ± 1 | 51 ± 2 | 9 (8, 10) |

Values are mean ± SD. ∆ = mean delta change, calculated as peak hypercapnia value minus baseline room air value, with 95% confidence intervals. HC: hypercapnia; P_ET_CO_2:_ partial pressure of end tidal carbon dioxide.

**Supplemental Table 2.** Physiological characteristics at baseline and after 3 minutes of continuous hypercapnia (HC) during acquisition of middle cerebral artery (MCA) images with MRI in obese pre-bariatric surgery patients, age-matched healthy controls, and young reference controls.

|  | **Obese pre-bariatric surgery** | | | **Age-matched healthy controls** | | | **Young reference controls** | | |
| --- | --- | --- | --- | --- | --- | --- | --- | --- | --- |
|  | **Baseline** | **HC** | **Δ (95% CI)** | **Baseline** | **HC** | **Δ (95% CI)** | **Baseline** | **HC** | **Δ (95% CI)** |
| Heart Rate (bpm) | 66 ± 6 | 67 ± 10 | 1 (−8, 8) | 60 ± 16 | 63 ± 16 | 3 (1, 5) | 53 ± 8 | 58 ± 7 | 5 (1, 10) |
| Breath rate (b/min) | 10 ± 4 | 13 ± 4 | 3 (1, 6) | 9 ± 4 | 11 ± 4 | 2 (1, 3) | 10 ± 3 | 12 ± 4 | 2 (1, 4) |
| P_ET_CO_2_ (mmHg) | 40 ± 4 | 49 ± 3 | 9 (6, 13) | 42 ± 6 | 51 ± 3 | 9 (6, 12) | 43 ± 3 | 50 ± 3 | 7 (6, 9) |
| MCA CSA (mm^2^) | 6.9 ± 0.7 | 6.7 ± 1.2 | −0.2 (−0.7, 0.1) | 6.8 ± 1.4 | 7.0 ± 1.4 | 0.2 (−0.3, 0.7) | 6.0 ± 1.1 | 6.7 ± 1.0 | 0.7 (0.2, 1.3) |

Values are mean ± SD. ∆ = mean delta change, calculated as hypercapnia value minus baseline room air value, with 95% confidence intervals. HC: hypercapnia; MCA: middle cerebral artery; CSA: cross-sectional area; P_ET_CO_2:_ partial pressure of end tidal carbon dioxide.

**Supplemental Table 3.** Physiological characteristics at baseline and after hypercapnia (HC) during acquisition of blood-oxygen-level-dependent (BOLD) images with MRI before bariatric surgery, and 2 weeks, and 14 weeks post-surgery.

|  | **Pre-surgery** | | | **2 weeks post-surgery** | | | **14 weeks post-surgery** | | |
| --- | --- | --- | --- | --- | --- | --- | --- | --- | --- |
|  | **Baseline** | **HC** | **Δ (95% CI)** | **Baseline** | **HC** | **Δ (95% CI)** | **Baseline** | **HC** | **Δ (95% CI)** |
| Heart Rate (bpm) | 69 ± 5 | 74 ± 6 | 5 (4, 6) | 68 ± 9 | 72 ± 10 | 4 (2, 7) | 65 ± 9 | 69 ± 9 | 4 (5, 12) |
| Breath rate (b/min) | 9 ± 4 | 13 ± 4 | 4 (1, 6) | 10 ± 4 | 13 ± 3 | 3 (1, 4) | 10 ± 4 | 12 ± 3 | 2 (−1, 6) |
| P_ET_CO_2_ (mmHg) | 39 ± 4 | 49 ± 3 | 10 (8, 11) | 38 ± 6 | 48 ± 5 | 10 (8, 11) | 40 ± 5 | 50 ± 3 | 10 (7, 13) |

Values are mean ± SD. ∆ = mean delta change, calculated as hypercapnia value minus baseline room air value, with 95% confidence intervals. HC: hypercapnia; P_ET_CO_2:_ partial pressure of end tidal carbon dioxide.

**Supplemental Table 4.** Physiological characteristics at baseline and after 3 minutes of continuous hypercapnia (HC) during acquisition of middle cerebral artery (MCA) images with MRI before bariatric surgery, and 2 weeks, and 14 weeks post-surgery.

|  | **Pre-surgery** | | | **2 weeks post-surgery** | | | **14 weeks post-surgery** | | |
| --- | --- | --- | --- | --- | --- | --- | --- | --- | --- |
|  | **Baseline** | **HC** | **Δ (95% CI)** | **Baseline** | **HC** | **Δ (95% CI)** | **Baseline** | **HC** | **Δ (95% CI)** |
| Heart Rate (bpm) | 66 ± 6 | 67 ± 9 | 1 (−8, 8) | 66 ± 10 | 66 ± 12 | 0 (−9, 9) | 61 ± 10 | 63 ± 7 | 2 (−7, 12) |
| Breath rate (b/min) | 10 ± 4 | 13 ± 4 | 3 (1, 6) | 11 ± 5 | 15 ± 5 | 4 (2, 5) | 10 ± 5 | 14 ± 5 | 4 (−4, 12) |
| P_ET_CO_2_ (mmHg) | 40 ± 4 | 49 ± 3 | 9 (6, 13) | 40 ± 6 | 51 ± 4 | 11 (8, 14) | 40 ± 5 | 51 ± 3 | 11 (7, 14) |
| MCA CSA (mm^2^) | 6.9 ± 0.7 | 6.7 ± 1.2 | −0.2 (−1.0, 0.7) | 6.7 ± 0.5 | 7.4 ± 0.5 | 0.7 (0.2, 1.3) | 7.0 ± 0.9 | 7.3 ± 1.1 | 0.3 (−0.3, 1.0) |

Values are mean ± SD. ∆ = mean delta change, calculated as hypercapnia value minus baseline room air value, with 95% confidence intervals. HC: hypercapnia; MCA: middle cerebral artery; CSA: cross-sectional area; P_ET_CO_2:_ partial pressure of end tidal carbon dioxide.

**Supplemental Figures**


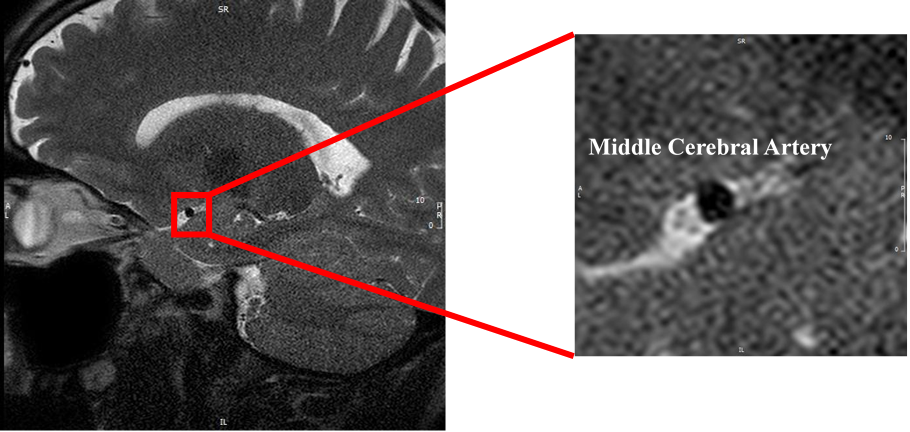


**Supplemental Figure 1.** Sagittal T2 baseline image (room air) of the left middle cerebral artery (MCA) of a representative subject. To assess MCA vasodilation capacity, the MCA cross-sectional area (CSA) was measured at baseline while subjects breathed room air and again after 3 min of hypercapnia (5% CO_2_, 21% O_2_, N_2_ balance) to calculate absolute and percent change in CSA.
